# Embracing change: practical and theoretical considerations for successful implementation of technology assisting upper limb training in stroke

**DOI:** 10.1186/1743-0003-9-52

**Published:** 2012-08-02

**Authors:** Ananda Hochstenbach-Waelen, Henk AM Seelen

**Affiliations:** 1 Adelante Centre of Expertise in Rehabilitation and Audiology, Zandbergsweg 111, 6432 CC, Hoensbroek, The Netherlands; 2 Department of Industrial Design, User Centred Engineering Group, Technical University Eindhoven, Den Dolech 2, 5600 MB, Eindhoven, The Netherlands; 3 Research School CAPHRI, Department of Rehabilitation Medicine, Maastricht University, Universiteitssingel 40, 6229 ER, Maastricht, The Netherlands

**Keywords:** Stroke, Brain injury, Rehabilitation, Upper limb, Upper extremity, Therapy, Technology, Implementation

## Abstract

**Background:**

Rehabilitation technology for upper limb training of stroke patients may play an important role as therapy tool in future, in order to meet the increasing therapy demand. Currently, implementation of this technology in the clinic remains low. This study aimed at identifying criteria and conditions that people, involved in development of such technology, should take into account to achieve a (more) successful implementation of the technology in the clinic.

**Methods:**

A literature search was performed in PubMed and IEEE databases, and semi-structured interviews with therapists in stroke rehabilitation were held, to identify criteria and conditions technology should meet to facilitate (implementation of) technology-assisted arm-hand skills training in rehabilitation therapy of stroke patients. In addition, an implementation strategy frequently applied in general health care was used to compose a stepwise guidance to facilitate successful implementation of this technology in therapy of stroke patients. Implementation-related criteria mentioned by therapists during the interviews were integrated in this guidance.

**Results:**

Results indicate that, related to therapy content, technology should facilitate repetition of task-related movements, tailored to the patient and patient’s goals, in a meaningful context. Variability and increasing levels of difficulty in exercises should be on offer. Regarding hardware and software design of technology, the system should facilitate quick familiarisation and be easily adjustable to individual patients during therapy by therapists (and assistants). The system should facilitate adaptation to individual patients’ needs and their progression over time, should be adjustable as to various task-related variables, should be able to provide instructions and feedback, and should be able to document patient’s progression. The implementation process of technology in the clinic is provided as a stepwise guidance that consists of five phases therapists have to go through. The guidance includes criteria and conditions that motivate therapists, and make it possible for them, to actually use technology in their daily clinical practice.

**Conclusions:**

The reported requirements are important as guidance for people involved in the development of rehabilitation technology for arm-hand therapy of stroke patients. The stepwise guide provides a tool for facilitating successful implementation of technology in clinical practice, thus meeting future therapy demand.

## Background

Stroke is the major cause of disability in the European Union [1]. Longitudinal studies of recovery after stroke revealed that only ~50 % of stroke survivors regain a functional arm [2,3]. For the remaining patients permanent sensory and/or motor disability of the hand constitute a major problem, since they experience difficulties to use the hand in functional activities of daily life [4]. In addition to the physical disabilities, social life is greatly affected [5,6]. A major determinant for the quality of life (QoL) of stroke patients is maintaining social relationships. The latter are influenced by social support, independence from others, and the ability to communicate [7]. In general, recovery of arm function after stroke is poor compared with recovery of leg function [6,8]. One of the reasons for this could be that after a stroke the arm is trained relatively little compared with the leg and trunk, since general mobility and the ability to transfer is prioritized over arm functionality. To improve use of the arm and hand of stroke patients in functional activities of daily life, new therapy strategies targeting recovery of arm function and arm-hand skill performance are needed.

The International Classification of Functioning, Disability and Health (ICF) [9,10] classifies health and disability at 1) impairment/function level, i.e. related to impairments in body functions and body structures; 2) activity level, i.e. related to limitations in executing activities; and 3) participation level, i.e. related to restrictions and problems in involvement in societal situations. In the choice for a therapy strategy, therapists should take into account that an individual’s health status relates not only to function level, but also to activity level and participation level of the individual. Previously, therapy predominantly addressed function level. However current scientific evidence shows that task-specific training, i.e. training of skills that are meaningful to the stroke patient, is essential in motor recovery of the upper limb in post-stroke rehabilitation [11-15]. Results of a systematic review [16] showed that impairment-oriented therapies failed to improve functionality, while task-oriented therapies did show improvement on the tasks that were trained. This indicates that, to improve arm-hand skill performance, it is necessary to direct upper limb therapy towards ICF activity level in addition to ICF function level.

Since stroke incidence in Europe is expected to increase from 1.1 million per year in 2000 to 1.5 million per year in 2025 [17], a general increase in the amount of therapy is foreseen. Rehabilitation technology could play an important role as therapy tool to meet this increased demand for therapy. Technology may be used to provide upper limb training, thereby decreasing the time a therapist needs to provide the training. In this way therapists are able to spend more time on treatment of other patients. In addition, technology-assisted therapy may create new possibilities, for example to offer upper limb training much earlier in the rehabilitation process and/or increase the amount of therapy of a patient.

During the last 15 years many devices have been developed that may assist in (training of) upper extremity movements [18,19]. However, although a number of devices have been tested regarding training efficacy, widespread application, or implementation, of the devices in the clinic remains low [20,21]. This implementation problem should be solved in order for technology to be actually applied more in (daily) rehabilitation routines. In medical care and rehabilitation, implementation is defined as the stepwise, systematic introduction of innovations and/or changes in care delivery that have been proven to be effective, with the purpose of structurally incorporating these innovations and/or changes in care delivery into professional performance of individuals, organizations and/or health care structures [22]. Since technology can play an important role as therapy tool in future training of arms and hands in stroke patients, the aforementioned implementation problem should be solved by identifying the causes that prevent implementation of technology in day-to-day clinical practice. First of all, reasons of therapists for (not) using technology in their therapy should be revealed. In addition, criteria and conditions that people, involved in the development of technology for arm-hand training, should take into account to achieve a (more) successful implementation of such technology in the clinic, should be identified. In this context, ‘criteria’ applies to specifications a device should meet to assist in upper limb therapy, whereas ‘conditions’ applies to device-related circumstances that should be fulfilled in order to be able to use the device in practice. The aim of the study that led to the current paper has been:

1) to provide an overview of criteria that technology (development) should meet to assist rehabilitation therapy aimed at arm-hand skills in stroke patients.

2) to provide an overview of criteria and conditions for implementation of the aforementioned technology in daily clinical practice.

3) to provide a stepwise guidance for people involved in technology development to facilitate successful implementation of technology-assisted arm-hand skills training in rehabilitation therapy of stroke patients.

The current review does not include clinical results on arm hand performance of 1) training concepts used in arm-hand rehabilitation, or 2) therapy with technology, since this has already been reported by other research groups [18,20,23-27].

## Methods

To obtain insight into why the implementation of available technology in the rehabilitation clinic lags behind, criteria and conditions, that are important in technology development towards a successful implementation in the clinic, should be identified. Firstly, criteria technology should meet to be useful and usable in assisting stroke rehabilitation therapy, should be identified. For technology to be ‘useful’, it should accommodate rules that govern therapy content (therapy-related criteria). ‘Usable’ applies to criteria related to hardware and software design of technology (software- and hardware-related criteria). Furthermore, criteria and conditions technology should meet to motivate therapists, and make it possible for them, to actually use the technology in their daily clinical practice (implementation-related criteria and conditions) should be identified. Two steps were undertaken to identify the aforementioned criteria and conditions, i.e. 1) a literature search was performed, and 2) semi-structured interviews [28] with therapists in stroke rehabilitation were held.

1) The literature search was carried out in PubMed and IEEE databases. The search was aimed at literature on the use of technology in upper limb rehabilitation of stroke patients. Combinations of MeSH terms and/or keywords used in the search strategy were the following: “Stroke”, “Upper Extremity”, “Self-Help Devices”, “Technology”, “Technology Assessment, Biomedical”, “Biomedical Technology”, and “Biomedical Enhancement” as MeSH terms; “clinical”, “acceptance”, “technology”, “stroke”, “rehabilitation”, “upper”, “limb”, “extremity” and “implementation” as keywords. In addition, articles marked as “related articles” in PubMed and reference lists of potential articles were screened. The search strategy identified approximately 950 articles. These 950 papers were subsequently screened whether they contained a) criteria that technology should meet in order to offer arm-hand training to stroke patients, and/or b) criteria and conditions for application of this technology in the clinic. The latter screening resulted in 9 eligible papers.

2) The semi-structured interviews were conducted among 6 senior physiotherapists and occupational therapists working in stroke rehabilitation and one physiotherapist working in children’s rehabilitation at Adelante Rehabilitation Centre in Hoensbroek, the Netherlands. On average they had more than 18 years of experience. The interviews were aimed at identifying the main reasons of therapists in deciding whether or not to use technology in their therapy. Especially the supporting and restricting aspects that influence this decision were to be identified. In addition, the interviews were directed at obtaining insight into a) criteria technology should meet to be useful and usable in assisting arm-hand skills training in stroke patients, and b) criteria and conditions for motivating therapists to use specific technological devices in their therapy, essential for a successful implementation of the device in the clinic.

A third step that was undertaken in the current study was aimed at providing a stepwise guidance to facilitate successful implementation of rehabilitation technology in therapy of stroke patients:

3) The strategy of Grol et al. [29,30] was used to compose this guidance. This implementation strategy is frequently applied during implementation of an innovation in health care in general. The guidance in the present paper is composed from a therapist’s perspective, by integrating the implementation-related criteria and conditions mentioned by therapists during the interviews.

The target group of people that has to adopt an innovation plays a major role in the implementation process. Regarding the implementation guidance, it should be mentioned that this target group is generally categorized into 5 subgroups, according to the ‘Diffusion of Innovations Theory’ of Rogers [30,31]:

a. ‘Innovators’ are the first subgroup of individuals that embrace an innovation. They are strongly focused on innovation, i.e. they are willing to bring about change. The latter is important for introduction of innovations.

b. ‘Early adopters’ are the second subgroup to adopt an innovation. They are highly respected within the target group and therefore are able to convince more people of embracing the innovation.

c. The ‘early majority’ is the third subgroup to adopt an innovation. They have close contact with and let themselves be guided by the ‘early adopters’. To convince this subgroup, the attitude of the ‘early adopters’ towards the innovation is important.

d. The ‘late majority’, the fourth subgroup, is sceptical to change and relies more on public opinion or pressure of colleagues. The latter may persuade them to adopt an innovation.

e. The ‘laggards’, the last subgroup, have strong resistance against change. They may adopt an innovation only after they are completely certain the innovation works.

## Results

In the first part of the results section the therapy-related and the software-/hardware-related criteria are described consecutively. The second part of the results section presents a stepwise guidance to facilitate successful implementation of technology in upper limb rehabilitation therapy, including a description of the implementation-related criteria and conditions. Criteria and conditions are represented from a ‘therapist’s perspective’, and have been identified through the interviews and literature search.

### Criteria rehabilitation technology should meet to be useful and usable in assisting arm-hand therapy in stroke patients

#### Therapy-related criteria

The senior physiotherapists and occupational therapists interviewed in the current study provided information on, among others, criteria that development of rehabilitation technology should meet to be useful in assisting arm-hand therapy in stroke patients. Results from these interviews largely corroborate findings of Timmermans et al. [19]. An overview of therapy-related criteria is presented in Table 1. 

**Table 1:** Therapy-related criteria technology should meet to assist in stroke rehabilitation therapy


a	be oriented at a patient’s goal(s) and his/her ability to accomplish these goal(s): training load should be patient-tailored and goal-tailored
b	take into consideration the individual cognitive impairments
c	be task-oriented and should resemble real life context of patients as close as possible; address both function, activity and participation levels of the ICF by offering strength training, task-oriented training and bilateral training; happen in a natural environmental context
d	be built up in stages of increased difficulty
e	offer variability in exercises
f	increase intensity and frequency of meaningful task-related movements
g	include feedback to the patient
h	be motivating and challenging for patients (inclusion of a gaming element)

Rehabilitation therapy uses personal goal setting of the patient, based on difficulties the patient experiences. A priority in therapy is reaching independence of the patient in achieving his or her goal(s). Therapists stated that *training should be oriented at a patient’s goal(s) and his/her ability to accomplish these goal(s)*. This includes that *training load should be patient-tailored and goal-tailored*[19].

Therapists stated that *training should take into consideration the individual cognitive impairments* which limit the ability of a patient to perform tasks. For instance, lack of spatial awareness may restrict the ability to dress; lack of awareness of the affected body side may hinder the use of the affected arm and hand. Technology-assisted training should, wherever possible and needed, take into account these impairments by accommodating patient-specific learning strategies that make task performance possible for the individual patient.

*Training should be task-oriented*, i.e. be directed at training the specific task or components of the task the patient wants to master again, *and should resemble real life context of patients as close as possible*, as was reported by therapists during the interviews. Specific movements that occur within the task should be trained in a meaningful context. For instance, if the goal is ‘learning to drink from a cup’, pronation-supination of the forearm is a movement necessary for emptying the cup in the mouth. Training the pronation-supination movement is meaningless, unless it is performed while holding a cup. Although e.g. strength, being a function level component, is fundamental for good skill performance, for optimal motor control *training should address both function, activity and participation levels of the ICF by offering strength training, task-oriented training and bilateral training*[19]. In addition, *training should happen in a natural environmental context*[19]. For robotic rehabilitation to be useful, the skills learned must transfer to real-world tasks by using real-world objects [20]. Furthermore, task training in a virtual reality environment has also been shown to be effective in translation to task performance in the real-world environment [20].

Basic functions (e.g. mobility, coordination, muscle force, sensibility) of the arm and hand underlie performance of upper limb movements, including task performance. A skill is learned in three consecutive phases [32]. In the first phase, i.e. the cognitive phase, cognitive activity is necessary to determine appropriate strategies to perform a new task. When the most effective strategy is determined, the second phase, i.e. the associative phase, begins: through subtle adjustments skill performance is improved. After a long time of practice the third phase, i.e. the autonomous phase, is entered: the task can be performed with less or no interference from other simultaneous activities [32]. To improve skill learning during these phases, therapists reported that *training should be built up in stages of increased difficulty*. Examples are: start training with a light object and progress to a heavy object; start training subtasks and progress to complete task performance; controlling the degrees-of-freedom, as explained in more detail in the results section ‘software- and hardware-related criteria’.

Therapists stated that *training should offer variability in exercises* to the patients, since, for example, this 1) keeps patients motivated and challenged, 2) improves generalisability of treatment effects, 3) improves retention of effects.

As reported by the therapists, and confirmed by literature [19], main principles of therapy are that *intensity* (i.e. number of repetitions per time unit/session) *and frequency* (i.e number of training sessions per day/week) *of meaningful task-related movements should be increased by training*. Technology could play an important role in this, especially if patients can train by themselves with the technology. As reported in literature on robotics, this aspect is a potential benefit of robotic rehabilitation [20,21,33].

Therapists stated that *training should include feedback to the patient* about their performance, tailored to the individual patient. Timmermans et al. [19] reported in depth on criteria for feedback on exercise performance: a) Knowledge of results (KR) and knowledge of performance (KP) should be available; b) Progress components of feedback that can contribute to motor learning are: i) fading frequency schedule, ii) from prescriptive to descriptive feedback, iii) from general to more specific feedback, iv) from simple to more complex feedback; c) Empty time slot for performance evaluation before and after giving feedback; d) Guided self-control on timing delivery feedback; and e) Feedback on error and correct performance.

Therapists reported that *training should be motivating and challenging for patients; inclusion of a gaming element* can contribute to both requirements. The following criteria are related to motivational aspects [19]: a) Training should include fun and gaming; b) The active role of the patient in rehabilitation should be stimulated by i) therapist independence on system use, ii) individual goal setting that is guided to be realistic, iii) self-control on delivery time of exercise instructions and by feedback that is guided to support motor learning, and iv) control in training protocol.

#### Software- and hardware-related criteria

An overview of software- and hardware-related criteria technology should meet to assist in stroke rehabilitation therapy is presented in Table 2.

**Table 2:** **Software- and hardware-related criteria technology should meet to assist in stroke rehabilitation therapy **^**1 **^


a	Familiarisation with the system should take little time^2^
b	Adjusting the hardware and setting the software to an individual patient should be a quick and easy process for all therapists and preferably also for therapy assistants^2^
c	Training goal(s) for patients of pre-programmed tasks or games should be evident to therapists^2^
d	Hardware and software design of technology should facilitate adaptation to individual patients or patient target groups and to patient progression over time^2^
e	Hardware and/or software settings should be adjustable to various task-related variables^2^
f	The design of the system (software and hardware) should facilitate the elicitation of task-related movements, whereas compensation strategies are to be prevented (as much as possible)^2^
g	The system should be able to give clear instructions, and provide feedback, to the patient^2^
h	The system should be able to measure and document task progression of the patient to provide performance feedback to the therapist^2^
i	Hardware and/or software should meet the implementation-related criteria: “The system should have quick initialisation, should preferably be portable and should function stably” ^2^
j	The system should be able to save individual therapy settings and data of a patient^2^
k	Hardware and software should facilitate independent use of the system by patients^2^: Training at home requires portability of the device [21]
l	Criteria related to psychological aspects of robotic devices [35]:
	The system should remain rather ’invisible’
	The system should look 'human-friendly' and behave accordingly
m	Criterion related to ergonomic and logistic aspects of robotic devices [35]:
	The robot set-up must be rather flexible to cope with different applications and situations

^1^ Criteria obtained from literature are indicated with their reference number.

^2^ Indication that criterion is obtained from the therapists that were interviewed in the current paper.

Since therapists are fully occupied with providing therapy to patients, each amount of time they need for familiarisation with technology reduces therapy time. Therapists stated that *familiarisation with the system should take little time* in the phase before application of technology in therapy.

During therapy, therapists will not use technology if set-up is too time-consuming, because it will limit individual therapy time that is typically scheduled for 30 minutes. Adjustable settings should be minimal. In addition, if only few therapists are specialized in the system, its use in therapy depends on them. This implies that the technology cannot be used if the technology-experienced therapists are not present or available, or that only the patients that receive therapy from the technology-experienced therapists are able to train with the system. Therapists reported that *adjusting the hardware and setting the software to an individual patient should be a quick and easy process for all therapists and preferably also for therapy assistants*. Ease of use is required [34], also because, in general, the technical background of users/therapists is limited [33,35]. If therapy assistants can set the system, this had two advantages. Firstly, application of an individual therapy program in a group setting, supervised by therapy assistants, is possible, and, subsequently, more people can be trained at the same time. Secondly, it allows an increase in intensity and frequency of therapy.

Therapists need to know what (sub)tasks and movements patients can train on with the exercises and games already programmed on the system. Therapists stated that, without much time investment, *training goal(s) for patients of pre-programmed tasks or games should be evident to therapists*. For example, a description of the training goal(s) may be included in the software.

*Hardware and software design of technology should facilitate adaptation to individual patients or patient target groups and to patient progression over time*, as stated by the therapists. This was also reported in literature as potential benefit of robotic rehabilitation [20]. This implies that the task, or the level of difficulty of a task, can be adapted to the needs and movement-related and cognitive abilities of a patient or patient target group. In this context patient target group refers to subgroups of patients that are classified on functional impairment level as being mildly, moderately or severely impaired. These subgroups represent respectively 1) patients with an arm hand that can be used for skill performance, 2) patients with low/beginning arm hand function, and 3) patients who have no functional arm hand performance. Lifting the arm and moving it to an object, i.e. reaching, is fundamental in grasping and picking up objects. The ability to reach and grasp depends on how much the arm and hand are affected. Controlling the degrees-of-freedom of the arm during reaching and grasping is a common method of therapists to increase or decrease the level of difficulty of a task; decreasing the degrees-of-freedom makes task performance easier. The therapy needs of the patient target group determine the requirements of technology development regarding this aspect. As to robotics, Lum et al. [21] reported that the possibility to provide active-assistive movement to more severely impaired patients may be beneficial. In addition to movement-related abilities, it should be possible to adapt hardware to the anthropometrics of a patient. Regarding robotic devices, the device should be adaptable to the human limb in terms of segment lengths, range of motion and degrees-of-freedom [35].

Therapists bring variation into exercises and tasks to increase e.g. the level of difficulty of tasks or intensity and frequency of training. Therapists reported that *hardware and/or software settings should be adjustable on various task-related variables.* Task-related variables therapists prefer to adjust are speed of task performance, task or movement repetitions, reaching area, and object variation. Adjustable settings of the system should be clear and meaningful to therapists.

During individual therapy, therapists pay attention to the quality of movement execution. Compensation strategies of a patient are minimized and controlled for by the therapist as much as possible to prevent patients from learning wrong movements. Regarding technology, the set-up of exercises depends largely on the design of the system. For training to be useful, therapists stated that *the design of the system (software and hardware)should facilitate the elicitation of task-related movements, whereas compensation strategies are to be prevented (as much as possible)*.

Therapists reported that *the system should be able to give clear instructions to the patient* about the exercise or task to be performed in a variety of ways, like an instruction movie, or verbal or written instructions. In this way instructions can be adapted to individual needs. Regarding robotics, in a study of Lee et al. [36] therapists reported that an animated simulation, that shows how an exercise is conducted with the robots in advance of starting the exercise, is preferred, but not necessary. Therapists in the current study stated that instructions should be directed at a) movement outcome, e.g. “pick up the cup and bring it to the mouth”, or b) movement performance, e.g. “stretch your arm, open your hand, grasp the cup, etc.”. In addition, therapists reported that *the system should be able to provide feedback to the patient* during or after an exercise, or after one or more exercise sessions. In this way motor learning can be improved. To be able to adapt to individual needs, variation in the way of providing feedback, e.g. verbal text, and variation in feedback content is preferred. Feedback content should include ‘feedback of performance’ (KP), i.e. movement performance, ‘feedback of results’ (KR), i.e. movement outcome, and/or feedback on progression within the exercise or training regarding amount of (un)completed repetitions. Feedback options should also include encouraging messages and sounds [36]. More details about what and how feedback can be provided were described earlier in the results section ‘therapy-related criteria’. To provide patient-specific therapy, therapists should be able to select if instructions/feedback should be given, when, how many times, in what way, and what kind of instructions/feedback.

Therapists reported that *the system should be able to measure and document task progression of the patient to provide performance feedback to the therapist.* In this way therapists are able to objectively assess task performance and possible progression of a patient and tailor therapy to the specific patient needs and abilities over time. Measurable variables are a) dosage of strength, e.g. how forceful is an object grasped and/or placed, b) speed and duration of movement or task performance, c) acceleration, d) coordination, e.g. trajectory of movement or deviation from intended trajectory, and e) selectivity, i.e. for example the ability to contract a muscle independent of contractions of other muscles. Compensation strategies can be controlled through feedback on performance of the movement. Currently, therapists observe these performance variables. Nevertheless, providing feedback on these variables with technology would be a big advantage for therapists, since it provides them with objective measurements which may be used to gauge therapy progression or effectiveness of therapy. Accurate measurement and tracking of patient’s impairments during a therapeutic intervention is a potential benefit of rehabilitation robots [37]. Riener et al. [35] reported that robotic devices should be able to measure patient's muscular effort and movement, to provide therapists with well-known scores for the evaluation of patient status and rehabilitation progress, e.g. Ashworth scale or Fugl-Meyer score. Parameters to register for documentation of the exercise history of a patient are: type of exercise, number of repetitions, speed of motion, range of motion, force of resistance and period of training [36].

In order to motivate therapists to use technology in therapy, *hardware and/or software should meet the implementation-related criteria: “The system should have quick initialisation, should preferably be portable and should function stably”*.

To prevent therapists from having to repetitively set the software each time they offer technology-related therapy to the patient, therapists stated that *the system should be able to save individual therapy settings and data of a patient.* This applies to both the exercises within a therapy program and saving the last condition trained on in the therapy program, in order to continue from that point onwards the next time. In this way a therapy program can be easily recalled, even without involvement of the therapist, making it possible for patients to quickly start training next time. Personal information of patients should be stored in an individual patient file [36]. Security measures should be installed to view patient data (personal information and performance data), e.g. requirement of user ID and password to access the data [36].

If patients are able to train by themselves in the rehabilitation centre or even at home, technology can play a major role in increasing therapy time, while minimizing therapist involvement. Therapists reported that *hardware and software should facilitate independent use of the system by patients*, i.e. the system should be easy to set up. Training at home requires *portability of the device*[21]. Training at home would make continued rehabilitation in chronic patients possible [20].

Criteria related to psychological aspects of robotic devices [35] are: a) *the system should remain rather ’invisible’*, i.e. it should not disturb the interaction between patient and therapist; b) *the system should look 'human-friendly' and behave accordingly*, i.e. it should be safe, as small and lightweight as possible, 'friendly looking', quiet and compliant. In other words, patients and therapists should not feel constrained by robotics [33]. Safety features for robotics are warning messages in case of emergency, an emergency stop button and/or an automatic machine stop and slow return to safe position after problem detection by the device [36].

A criterion related to ergonomic and logistic aspects of robotic devices is that *the robot set-up must be rather flexible to cope with different applications and situations*[35], e.g. different body heights and weights or additional equipment accompanying the patient like a wheelchair or respiratory equipment.

### A stepwise guidance, including implementation-related criteria and conditions, to facilitate successful implementation of technology in therapy

The implementation-related criteria and conditions mentioned by therapists during the interviews are integrated in the frequently applied implementation concept of Grol et al. [30], who consider implementation as a process of change that consists of five phases care givers have to go through. In this way guidance is provided in steps to be taken to facilitate successful implementation of rehabilitation technology in therapy of stroke patients.

#### Phase 1: Orientation

This phase is about creating awareness, interest and support for the new technology [30]. End-users, i.e. therapists (and assistants) working in stroke rehabilitation, should be made aware of the existence of the new device without infringing their therapy time. Information about the potential (e.g. device-assisted therapy goals), added value and advantages of the device for therapists and patients, e.g. increasing training frequency and intensity, may trigger their interest and support for the new technology.

#### Phase 2: Insight

This phase is about providing knowledge and understanding about use of the new technology, about insight into own conventional therapy methods and differences compared with technology-assisted therapy, and about the possible benefits of the new technology [30]. For this phase the following implementation-related conditions were identified:

· To start using a device in therapy, therapists require *insight into the therapy applicability of the technology* through *informative and practical courses/presentations.*

· A *short manual* on the device applications can be helpful.

Information provided should cover e.g. hardware and software use, potential patient group(s), device-assisted therapy components or goals, performable exercises, indications, contra-indications, etc.

Managers should arrange time for therapists to attend these courses and/or presentations, otherwise therapists will choose for their first priority, i.e. providing therapy to patients.

#### Phase 3: Acceptance

This phase is about creating a positive attitude, motivation and willingness to change [30]. For acceptance of new technology the following implementation-related criteria and conditions should be met:

· *The device should fit within the vision of the current stroke rehabilitation*; i.e. the device should fit within the current general vision on how to provide therapy and training to people after they suffered from a stroke, in order to improve their recovery process. This implies that technology should meet the therapy-related criteria, like e.g. increasing intensity and frequency of useful training.

· *Therapists should be convinced of the added value and effectiveness of the technology*, as a result of:

a. obtained scientific evidence on training effects of the system, i.e. obtained from clinical research [34,37];

b. observations that the patient improves on clinimetrics, reported by Lum et al. [21] as “the device must provide quantifiable, functional benefits to the patient”;

c. observations that the patient likes to train with the system.

· *Therapist and patient should already be (intrinsically) motivated to use the technology*.

· *The device should make it possible to treat more patients simultaneously*.

This is especially interesting from a managerial point of view. To meet this criterion, *a significant segment of therapy should be automated*[20,21]. This includes automation of interventions that are extremely physically demanding for therapists [37]. Fasoli et al. [38] reported on several benefits of robotics that are not easily achieved by additional conventional therapy, which may increase clinical acceptance: 

a. automated practice of repetitive movements;

b. programmable levels of assistive or resistive training to meet a variety of patient needs;

c. objective evaluation of motor abilities, e.g. speed, strength, range of motion, smoothness;

d. providing specific feedback (KR or KP) through sensing capabilities of robotics.

To create acceptance, (dis) advantages of the system should be clear to therapists in order to convince them about the usefulness of the device in practice [30]. Advantages are for example: effectiveness, less time consumption, increased training intensity and frequency. Therapists should be enabled by managers to work with the system. Acceptance is also related to emotions of therapists towards technology. For example, fear that technology could replace them, although the expertise of a therapist is necessary to determine patient’s individual needs [37]. Therapists interviewed in the current paper mentioned that, within the target group of therapists, it is necessary to primarily convince so-called ‘innovators’. If this subgroup is convinced, they can try to convince others, e.g. the ‘early adopters’ and the ‘early majority’. If the device is intended to be used in therapy, therapists should consider how to arrange this, to be discussed in meetings with fellow therapists (and managers). Involving therapists in the plan of action for use of the device in practice may increase the use of the device afterwards.

#### Phase 4: Change

This phase is about introducing the device in practice and confirming its usefulness [30]. Therapists should get the opportunity to work with the device and gain experience. For this phase the following implementation-related criteria and conditions have to be met:

·*The organization should create possibilities for therapists to familiarise themselves with the technology*. This implies:

a. creating time to work with the system over a longer period of time next to standard therapy time, e.g. practice together during team meetings of therapists;

b. making it possible to follow workshops about setting the software and hardware of the system to the individual patient’s needs.

When therapists have become familiar with the system, they should be enabled to practice with patients.

· *The device should be installed ‘ready-to-go’.* This implies that:

a. it should be able to start immediately, without excessive start-up procedures;

b. it should be easy to operate;

c. it should preferably be portable.

In this way, loss of individual therapy time is minimized. Portability of the complete system assures that patients can train in nearly any room, which, for example, is necessary for patients who are easily distracted and therefore require a separate quiet room to train.

· *The system should function stably (no failure)*.

Otherwise therapists will not continue to use it, nor do patients for that matter.

· *The device should complement individual therapy and/or be easy applicable in group therapy*.

The device has an added value if it can be used as additional therapy [21]. In individual therapy it will only be used if it has an additional value, which cannot be realized by the therapist [20,21].

After therapists have become familiar with the system and gained enough experience, evaluations should be planned with therapists and managers about the usability of the device, and the possibility to apply the device in practice without major problems, costs or damage. If in this phase of the implementation process problems have arisen, possible solutions should be considered. Based on their experiences, therapists should intrinsically come to the conclusion that applicability of the device is possible without major problems [30].

#### Phase 5: Retention of change

The last phase of implementation is about incorporation of the technology in existing practices and into the organization [30]. A plan of action should be set up, considering organizational, financial, and structural conditions that should be fulfilled for continuous application of the device in practice. In addition, the device-assisted therapy should be included in existing health care protocols. From a therapist’s perspective, the following implementation-related conditions were identified for this phase:

· *System should be placed in an easy accessible room, preferably the therapy room.*

· *Availability of the device should be clear for therapists.*

· *System should be frequently used in daily practice*.

## Discussion

The first aim of the present paper was to provide an overview of criteria that technology (development) should meet to assist rehabilitation therapy aimed at arm-hand skills in stroke patients. The second aim was to provide an overview of criteria and conditions for implementation of the aforementioned technology in daily clinical practice. The last aim was to provide a stepwise guidance for people involved in technology development to facilitate successful implementation of technology-assisted arm-hand skills training in rehabilitation therapy of stroke patients. The implementation-related criteria and conditions were combined with this stepwise guidance. The identified criteria and the guidance for implementation are reported from a therapist’s perspective.

Although much technology has been developed in the field of upper limb stroke rehabilitation, successful implementation of the devices in clinical practice still remains low. People involved in technology development should be aware that the use of technology in therapy largely depends on therapists. Since therapists decide themselves whether or not to use technology in their daily practice, technology should meet their wishes and needs in providing arm-hand therapy to stroke patients. Very little literature is available that reports on requirements for upper limb rehabilitation technology. During development of new rehabilitation technology for arm-hand therapy of stroke patients, the criteria and conditions reported in the present paper are important to consider. These criteria and conditions provide insight into which device specifications and device-related circumstances are essential to therapists for them to use a device in therapy.

A first step is to consider therapy-related and software- and hardware-related criteria, since these criteria determine if technology is useful and usable in therapy. Implementation may be improved if technology is developed according to these needs/criteria. In conjunction with this, involving therapists, and in a later stage also patients, as early as possible in the development phases of new technology can contribute to useful system development and successful implementation. The criteria reported in the current article are obtained from a therapist’s perspective. A close cooperation with therapists during development of specific technology may contribute to the understanding of developers as to what therapists explicitly need from technology for it to be useful and usable in assisting their therapy. Feedback of end-users on prototype specifications can be used to improve the device according to the needs of therapists and patients. This concept was applied in the development of the iPAM system, a dual-arm pneumatically actuated robot system designed to provide assisted active arm exercise for people with stroke [39]. End-users were involved through regular meetings in which progress was assessed and information on aspects of the design was provided by interactive demonstrations, informal discussions and questionnaires. In addition, end-users participated in exploratory studies during early phases of development, e.g. in an end-user acceptability study. Jackson et al. [39] reported that the feedback of end-users was invaluable in the development of the iPAM system and for future design improvements. This also applied to the interface development of the system [40].

In the next step the criteria and conditions that are important to start using the technology in clinical practice, i.e. implementation-related criteria and conditions, should be considered. This step may partly overlap with the previous one. One aspect of implementation is acceptance of the technology by therapists (and patients) [20], which depends on several criteria and conditions as reported in phase 3 of the stepwise guidance. The therapists interviewed in the present paper mentioned scientific evidence on effectiveness of the technology as one condition for acceptance of technology in clinical practice. To determine effectiveness, firstly a final version of the technology should be tested under controlled conditions with a small group of end-users in so-called "user trials". If these small trials show that the technology is useful and usable in therapy, randomized clinical trials should be performed, in which effects of conventional therapy are compared with effects of technology-assisted therapy in stroke patients. To determine which group(s) of stroke patients may benefit most, a broad range of patients with different functional levels regarding arm hand performance should be included. Results from these randomized clinical trials should be used to assess effectiveness of the new technology. Backus et al. [41] provided therapists with tools to critically appraise the available literature on efficacy-related evidence and, consequently, to implement the evidence in clinical practice. They reported that, at first, the validity of the evidence should be determined. If validity is determined, benefits and barriers of the intervention or technology should be identified and aspects of the evidence that are applicable and relevant to practice and patients should be determined. If the therapist is convinced about use of the technology in clinical practice, other stakeholders, like clients and caregivers, institution or organization, administration and financial stakeholders, should be extensively informed about the obtained information on the technology, in a meaningful context to them [41].

From a therapist’s perspective, proof of effectiveness of technology contributes to use in daily practice. Additionally, from the perspective of financial stakeholders cost-effectiveness is considered to be important. Technology-related costs remain a barrier in clinical application [20,37]. These costs include 1) equipment purchase, 2) time of staff, i.e. therapists and therapy assistants, needed to be trained to use the technology, and 3) staff time needed to set up the therapy program, set up the patients and monitor patients [20,37]. It is uncertain if staff time needed for technology-assisted therapy can be billed and will be paid for by insurance companies [20]. Also, cost-effectiveness of technology should be considered from a managerial or institutional point of view. A device should be affordable for clinics yet profitable for manufacturers and a device should not increase the costs of health care [21]. An effective device may even decrease the health care costs in two ways [21]: 1) if use of the device can speed up functional recovery, this consequently can reduce individual therapy time and in this way reduce therapy costs, 2) if use of the device can achieve a higher level of functional independence of patient, costs of long-term care may be reduced. To be affordable for clinics, low cost devices are preferred, if they are able to meet criteria and conditions for assisting rehabilitation therapy on arm-hand skills of stroke patients. For an organization to purchase new technology, cost-effectiveness should be assessed. Jones et al. [34] reported on a committee that was established to systematically review prospective technology to determine potential therapeutic applicability and overall benefit to the concerning organization. During this committee review clinical application and relevance, financial feasibility, marketability and safety are examined and discussed. In this way the potential value of new technology for use in clinical practice can be assessed, which can be used as guidance in purchasing the technology or not. A concept like the one reported by Jones et al. [34] can be beneficial for organizations to apply in future.

In addition to scientific evidence on effectiveness, the therapists interviewed in the present paper mentioned a second condition that is related to phase 3 of the stepwise guidance (acceptance of technology); that is the device should make it possible to treat more patients at the same time, thereby optimizing therapy time. One possibility is that the device(s) facilitates repetitive practice, while therapists can treat other patients at the same time [21]. Another possibility is the placement of several technological devices in one room in order to provide individual therapy sessions within a group therapy concept. The latter possibility also meets the condition reported in phase 4 of the stepwise guidance, namely ‘the device should be easy applicable in group therapy’. Buschfort et al. [42] studied the acceptance, utilization and first clinical results of an arm studio designed to intensify treatment of patients with a moderately to severely affected arm after stroke. The studio comprised 6 devices for either finger training or for bilateral training of selected distal and proximal arm movements. The majority of patients regarded the training positively. To run a 30–45 min group session of maximal 6 patients, only 1 therapist and 1 helper were needed. Although no conclusions on effectiveness could be drawn, this arm studio may be more cost-effective than additional individual therapy sessions [42]. Since therapy demand increases, a concept like this might be a promising way of providing (additional) therapy in future if effectiveness of the technology is warranted.

In a study of Dijkers et al. [43], therapists mentioned another aspect for making purchase of expensive technology worthwhile, i.e. further development of the technology towards usability in patients with different diagnoses. Therefore additional exercise programs should be developed that could be used by e.g. spinal cord injury or cerebral paresis. However, every diagnosis group has its own requirements the technology should meet. Technology should be adaptable to these requirements. This implies that the requirements for other diagnosis groups should be known and reported in that people involved in technology development can take these into account during development of a new device.

## Conclusions

The reported requirements in the present paper may be helpful for people involved in technology development as guidance in the development of rehabilitation technology for arm-hand therapy of stroke patients. Technology development according to needs and wishes of therapists is important for use of the technology in therapy in a later stage. Feedback of end-users during the development phases may contribute to improvement of a device. To facilitate a successful implementation of useful and usable technology in clinical practice, the reported guide, including implementation-related criteria and conditions from a therapist’s perspective, may be helpful. From an institutional point of view, proof of cost-effectiveness and the possibility to treat more patients at the same time are points to consider in the decision to purchase new technology. Although a lot of issues need to be taken into account during development of new technology, technology could play an important role in meeting the increasing therapy demand in future.

## Abbreviations

QoL: Quality of Life; ICF: the International Classification of Functioning, Disability and Health; KR: Knowledge of Results; KP: Knowledge of Performance.

## Competing interests

The authors declare that they have no competing interests.

## Authors’ contributions

AH-W and HAMS have made substantial contributions to conception and design of the manuscript, have been involved in drafting it and critically revising it for important intellectual content, and have given final approval for publication.
